# Supplementary material for: Structures of tweety homolog proteins TTYH2 and TTYH3 reveal a Ca2+-dependent switch from intra- to intermembrane dimerization
Source: Nat Commun. 2021 Nov 25;12:6913. doi: 10.1038/s41467-021-27283-8 (PMC8617170; doi:10.1038/s41467-021-27283-8)
Supplement: Supplementary file 1 — Supplementary Information [file 41467_2021_27283_MOESM1_ESM.pdf]

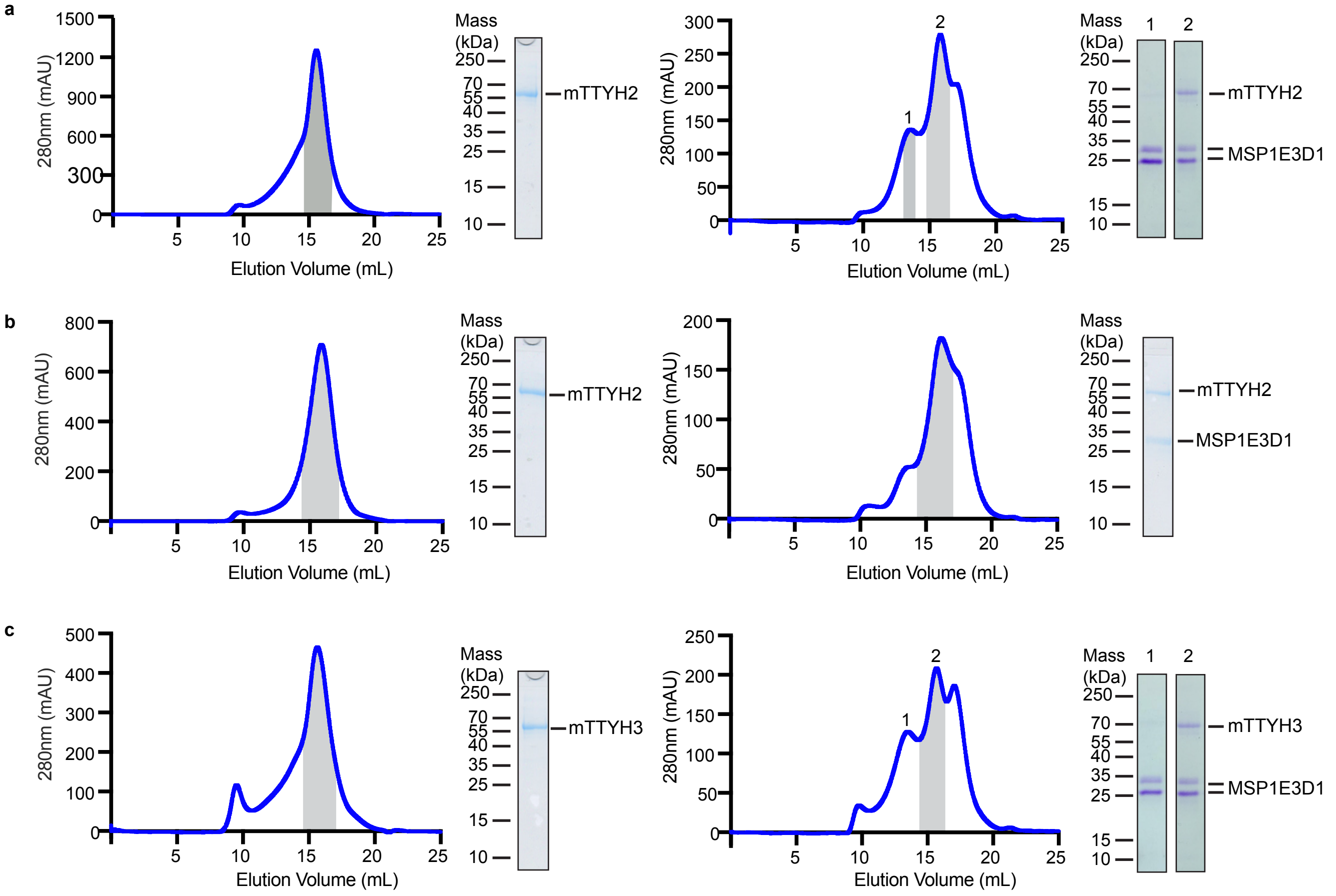

Supplementary Figure 1. Purification and lipid nanodisc reconstitution of TTYHs.
(a) Purification and reconstitution of TTYH2 in the presence of 1 mM Ca^{2+}. Size exclusion chromatogram of TTYH2 purified in DDM/CHS detergent and 1 mM Ca^{2+} (left) and reconstituted in MSP1E3D1 lipid nanodiscs (right). Pooled fractions are highlighted with corresponding Coomassie-stained SDS-PAGE of the final sample is shown. Early eluting peak 1 correponds to TTYH2-free MSP1E3D1 containing liposomes or aggregates. (b) Purification and reconstitution of TTYH2 in the absence of Ca^{2+}. Size exclusion chromatogram of TTYH2 purified in DDM/CHS detergent (left) and reconstituted in MSP1E3D1 lipid nanodiscs (right). Pooled fractions are highlighted with corresponding Coomassie-stained SDS-PAGE of the final sample is shown. (c) Purification and reconstitution of TTYH3 in the presence of 1 mM Ca^{2+}. Size exclusion chromatogram of TTYH3 purified in DDM/CHS detergent and 1 mM Ca^{2+} (left) and reconstituted in MSP1E3D1 lipid nanodiscs (right). Pooled fractions are highlighted with corresponding Coomassie-stained SDS-PAGE of the final sample is shown. Early eluting peak 1 correponds to TTYH3-free MSP1E3D1 containing liposomes or aggregates. Samples were run on a Superose 6 column.

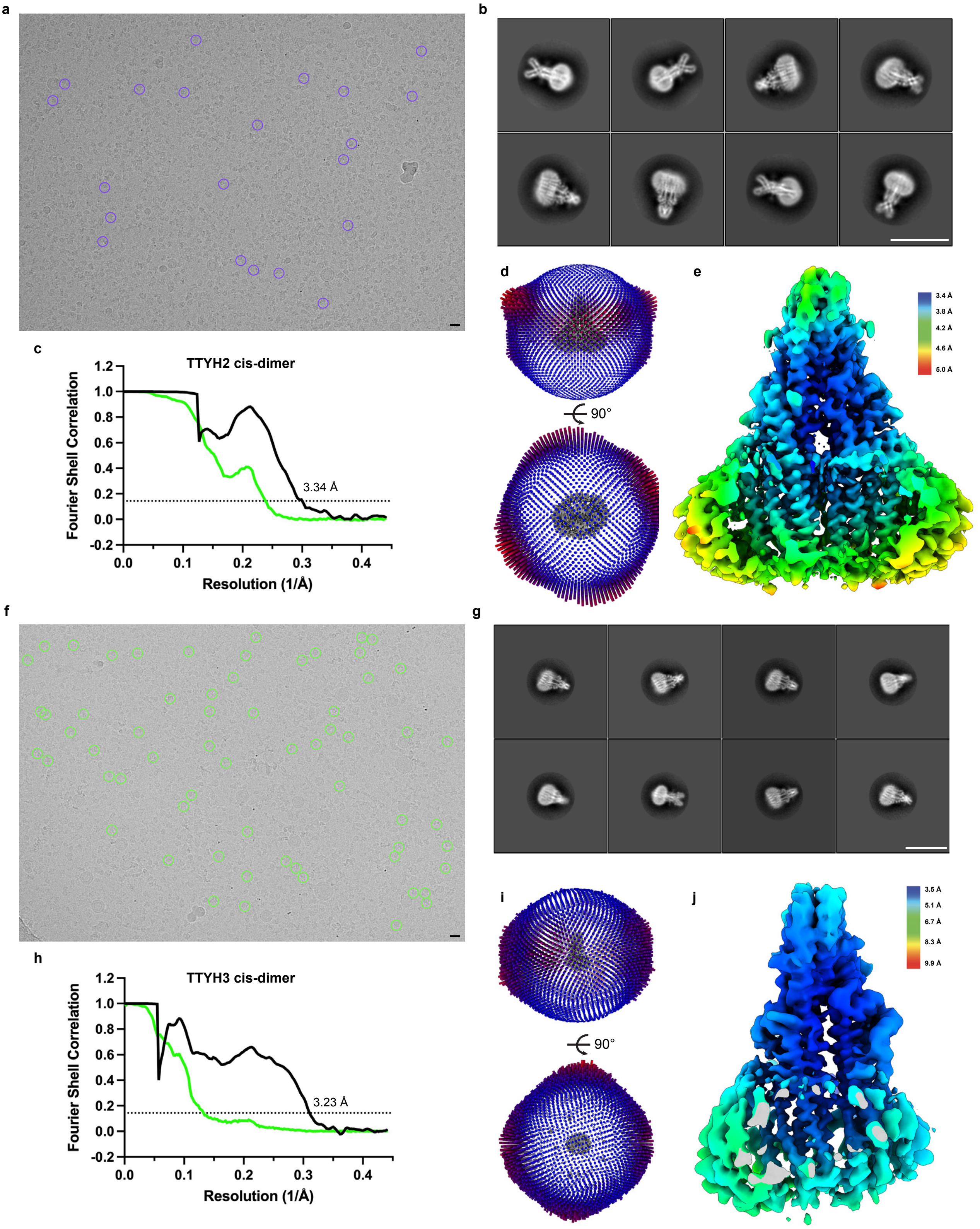

Supplementary Figure 2. Example micrographs, 2D class averages, and validation for TTYH2 and TTYH3 cis-dimers in the presence of Ca^{2+}. (a,f) Representative micrographs (of 5,014 and 3,217 total micrographs, respectively, (b,g) selected 2D class averages, (c,h) Fourier shell correlations between the two corrected (black) and unmasked (green) half maps, (d,i) angular distribution of particles used in final refinement and (e,j) Relion-estimated local resolution colored on the final maps of (a-e) TTYH2 and (f-j) TTYH3 cis-dimers determined in the presence of 1 mM Ca^{2+}. Scale bars are 150 Å.

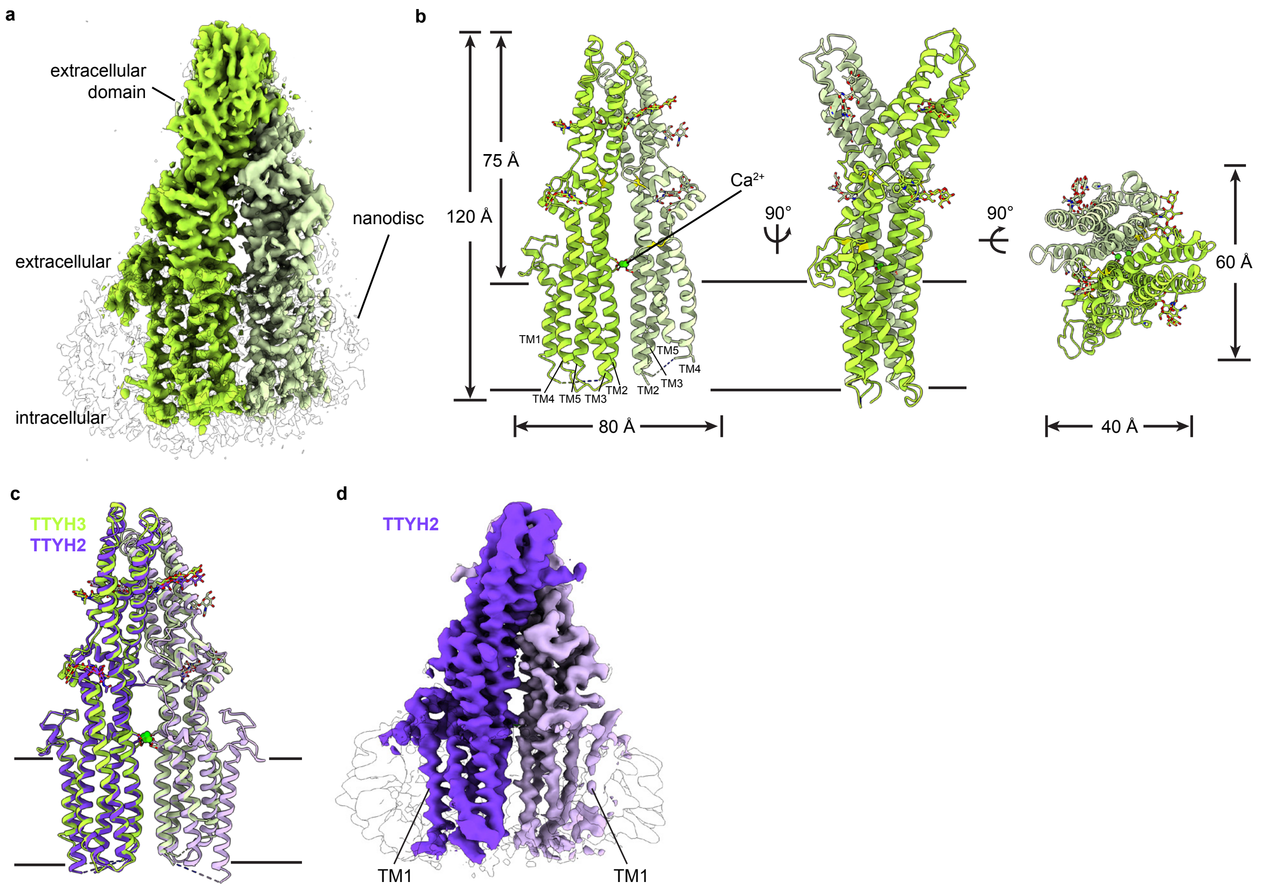

Supplementary Figure 3. Structure of a TTYH3 cis-dimer in the presence of Ca^{2+}.
(a) Cryo-EM map of a TTYH3 cis-dimer in MSP1E3D1 nanodiscs at 3.2 Å resolution viewed from the membrane plane. Density from one TTYH3 protomer is colored dark green, the second protomer is light green, and the nanodisc is white. (b) Model of the TTYH3 cis-dimer viewed from the membrane and from the extracellular side. N-linked glycosylation sites and disulfide bonds in the extracellular domain are drawn as sticks and Ca^{2+} ions are shown as green spheres. (c) Overlay of TTYH3 (greens) and TTYH2 (purples) cis-dimer models. TM1 is modeled in the both TTYH2 protomers, but is not modeled in the light green TTYH3 protomer. (d) Cryo-EM reconstruction from a subset of TTYH2 particles with an asymmetric nanodisc in which density corresponding to TM1 from the light purple protomer is poorly resolved and the neighboring disc density is weaker as observed in the TTYH3 reconstruction.

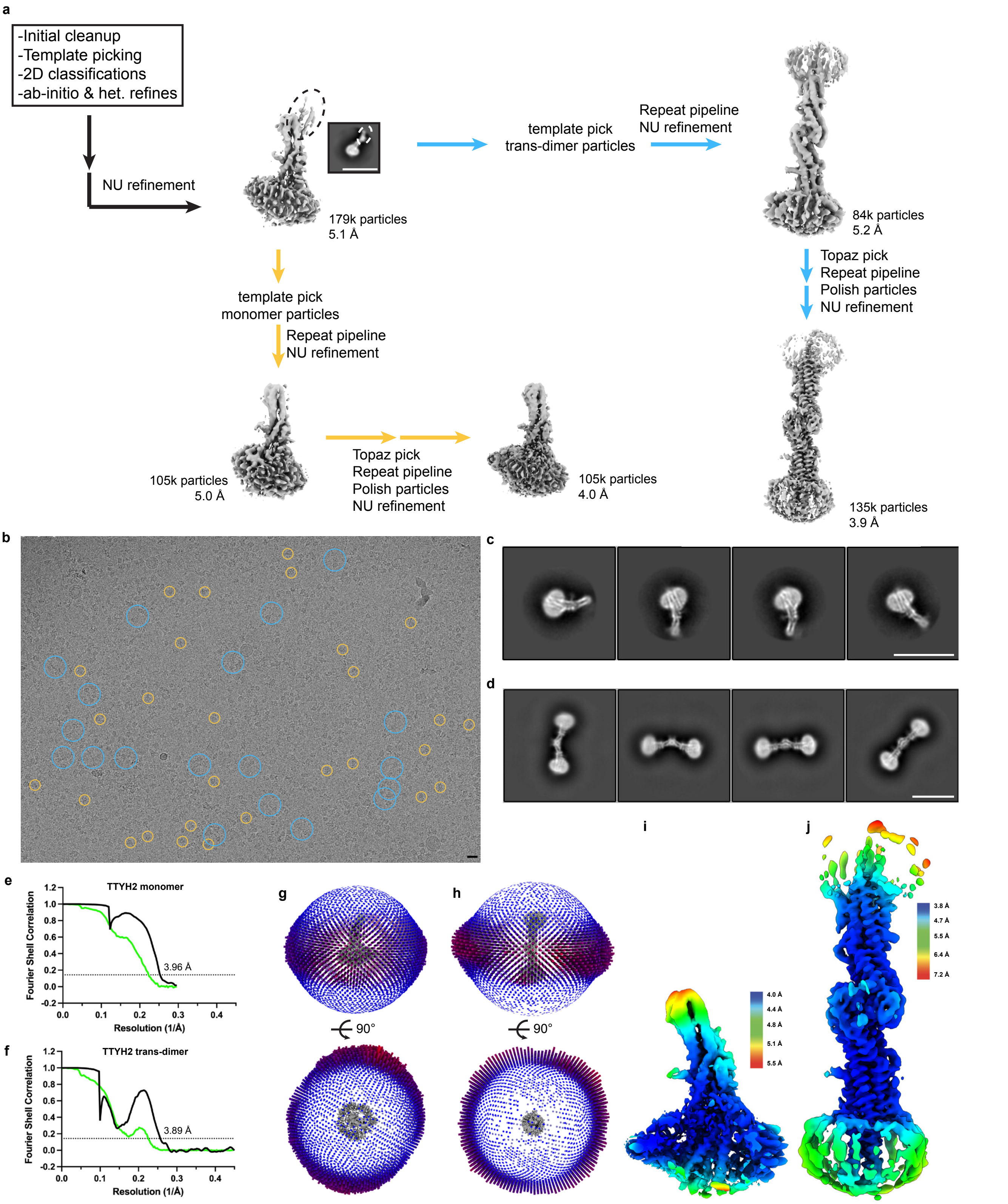

Supplementary Figure 4. Cryo-EM data processing and validation for TTYH2 monomers and trans-dimers in the absence of Ca^{2+}.
(a) Overview of data processing pipeline in cryoSPARC and Relion. Extra density at the distal end of the extracellular domain observed early in processing (circled with dashed lines) suggested the presence of trans-dimers in the dataset. (b) Representative micrograph (of 7245 total micrographs) with trans-dimers circled in blue and monomers circled in orange. (c) Selected 2D class averages of monomers and (d) trans-dimers. (e,f) Fourier shell correlations between the two corrected (black) and unmasked (green) half maps. (g) angular distribution of particles used in final monomer and (h) trans-dimer refinements. (i) Relion-estimated local resolution colored on the final monomer and (j) trans-dimer maps. Scale bars are 150 Å.

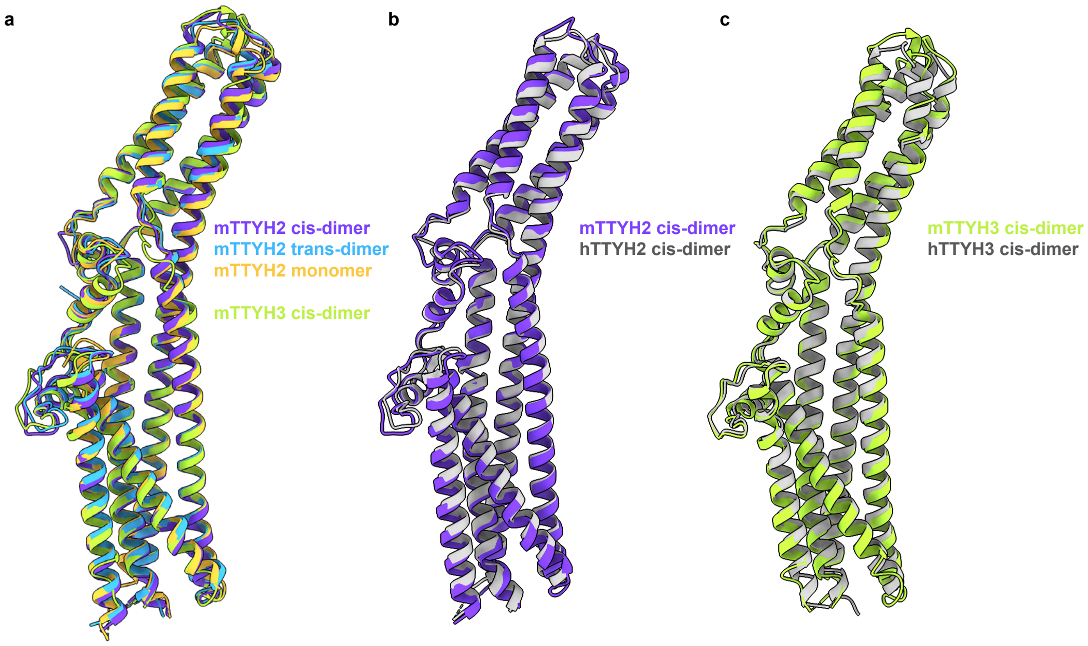

Supplementary Figure 5. Comparison of TTYH protomers from different structures.
(a) Overlay of the mTTYH2 monomer (orange, PDB: 7RTV; r.m.s.d. = 1.3 Å), one protomer from the mTTYH2 cis-dimer (purple, PDB: 7RTT), one protomer from the mTTYH3 cis-dimer (green, PDB: 7RTW; r.m.s.d. = 1.4 Å), and one protomer from the mTTYH2 trans-dimer (blue, PDB: 7RTU; r.m.s.d. = 1.5 Å). r.m.s.d.s are between protomers of the indicated structure and the mTTYH2 cis-dimer. (b) Overlay of one protomer from the mTTYH2 cis-dimer (purple, PDB: 7RTT) and hTTYH2 cis-dimer (gray, PDB: 7P54; r.m.s.d. = 1.2 Å). (c) Overlay of one protomer from the mTTYH3 cis-dimer (purple, PDB: 7RTW) and hTTYH3 cis-dimer (gray, PDB: 7P5C; r.m.s.d. = 1.2 Å). Large scale conformational changes are not observed between the structures.

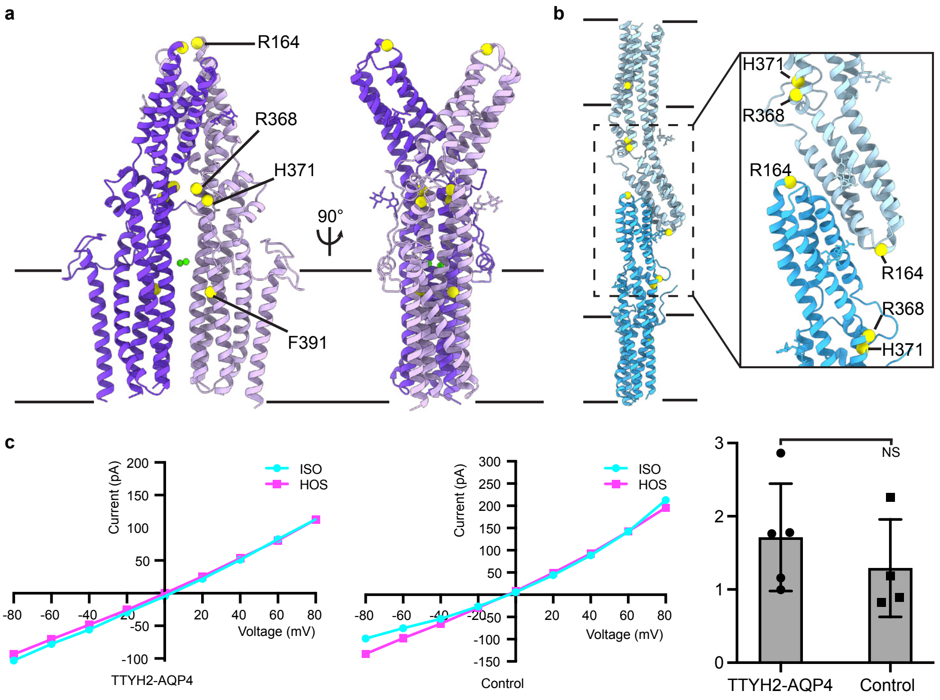

Supplementary Figure 6. Mutations previously reported to impact channel activity and lack of TTYH2-dependent currents in heterologous cells.
(a) TTYH2 cis-dimer and (b) TTYH2 trans-dimer viewed from the membrane plane with positions of point mutations previously reported to impact TTYH-dependent channel activity indicated with yellow spheres. (c) Representative current-voltage relationships and maximum fold-activation at 80 mV recorded in response to hypotonic extracellular solution-induced cell swelling (HOS). No significant difference between TTYH2 and AQP4 co-expressing cells and control cells is observed (mean ± sem, n=5 and 4 cells for TTYH2/AQP4 co-expressing and control cells, respectively, two-tailed p=0.41, unpaired Student's t-test).

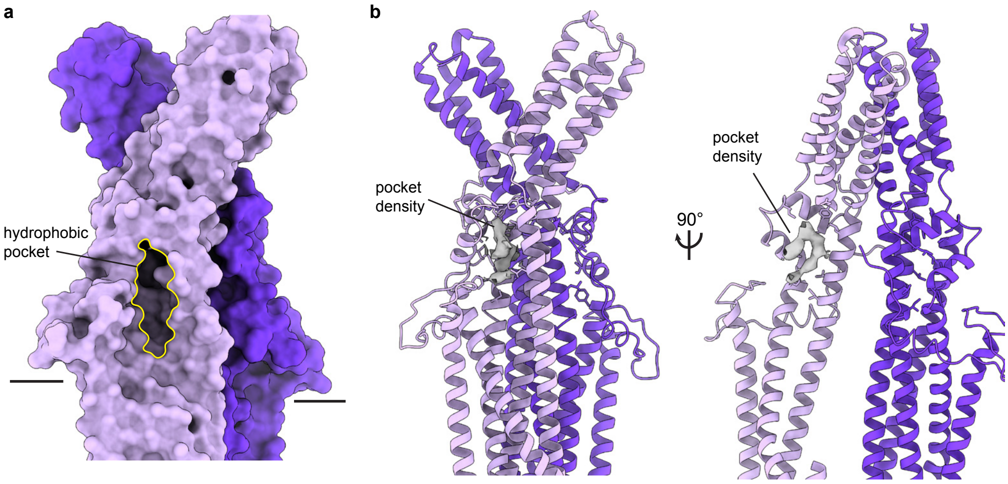

Supplementary Figure 7. A membrane-proximal hydrophobic pocket in the extracellular domain of TTYHs.
(a) TTYH2 cis-dimer molecular surface shown from the membrane plane. The hydrophobic pocket is outlined in yellow. (b) TTYH2 model with unassigned horseshoe-shaped pocket density indicated and hydrophobic residues lining the pocket shown as sticks.

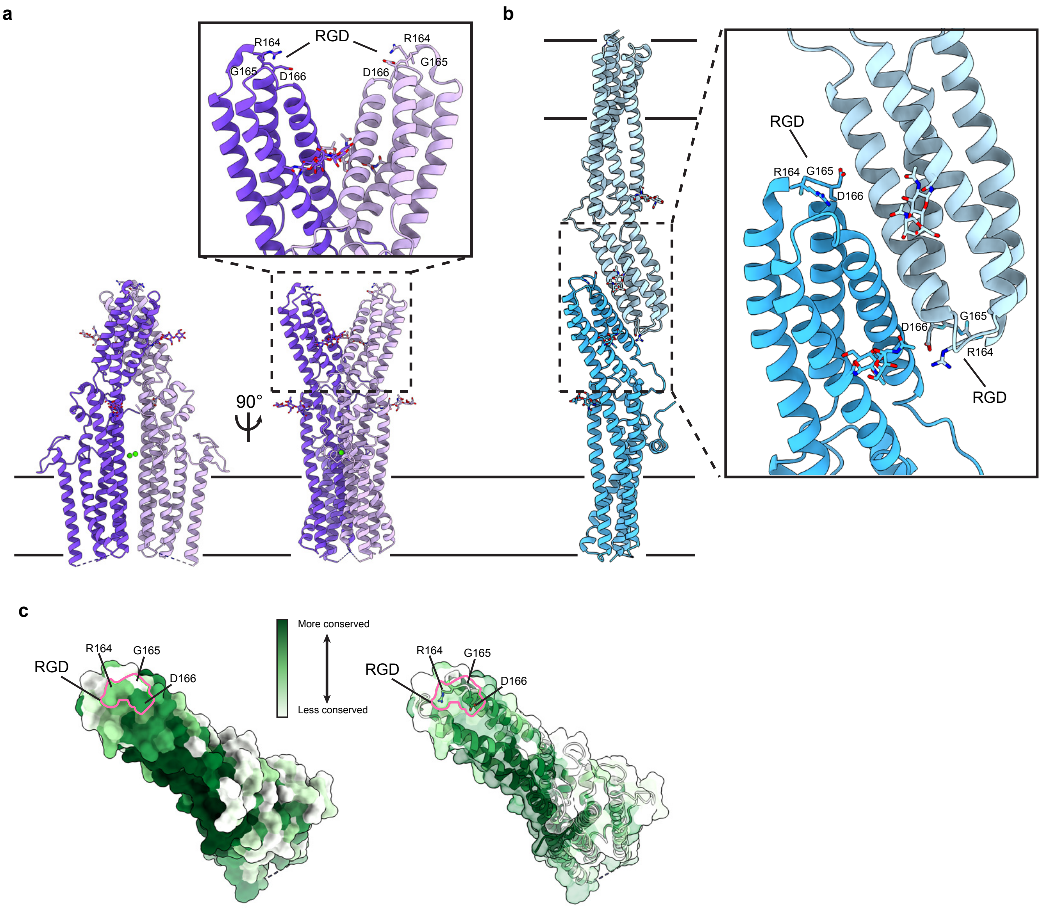

Supplementary Figure 8. An RGD motif at the distal tip of the TTYH2 extracellular domain.
(a) TTYH2 cis-dimer and (b) trans-dimer with the RGD motifs within the ED1-ED2 linker drawn as sticks. The RGD motifs are solvent-exposed in cis-dimers and monomers and partially occluded in trans-dimers at the interface between protomers. (C) TTYH2 surface viewed from above and colored according to conservation among chordate TTYH1-3 sequences. An opaque surface (left) and transparent surface view (right) are shown with the RGD motif outlined in pink and position of each residue indicated.

Supplementary Table 1. Codon optimized gene sequences

mTTYH2:

```
ATGCCAGCTGCACGCGTGGAATATATCGCACCTTGGTGGGTTGTGTGGTTGCACTCCGTTCCTCATTTGGGCCTGCGACTGCAGAGAGTGGAT
TCTACCTTTAGTCCTGGAGACGAAACCTATCAAGAGAGCCTCCTCTTCTTGGGAGTACTCGCAGCCATTGGTCTCGGATTGAATCTTATATTTTT
GACCGTATATCTCGTTTGCACTTGCTGTTGTAGGCGCGATCACACAGTTCAAACAAAACAACAGGAAAGCTGCTGTGTTACCTGGACTGCTGTG
GTGGCTGGCCTGCTGTGTTGCGCAGCTGTCGGGGTAGGATTCTATGGAAACTCAGAAACCAACGACGGGATGCACCAACTCATATATAGTCTG
GACAACGCTAACCACACATTCAGCGGTATGGATGAATTGGTTTCAGCAAACACTCAGAGGATGAAAGTCGATCTTGAACAACATCTCGCTAGGC
TGTCAGAGATCATAGCAGCAAGGGGCGATTATATCCAAACTCTCAAGTTTATGCAGCAGATGGCAGGGAACGTTGTTTCACAGTTGTCCGGCTT
GCCCGTGTGGAGAGAGGTCACCACCCAGTTGACCAAGCTGTCTCATCAAACAGCATACGTTGAGTACTATAGGTGGCTTAGTTACCTTCTTCTT
TTTATACTTGATCTCGTCATTTGTCTCGTCACTTGCCTGGGGCTGGCCCGACGCTCAAAATGTCTGTTGGCCAGCATGCTGTGTTGCGGGATAC
TGACATTGATTTTGAGCTGGGCTTCTCTGGCCGCCGACGCTGCCGCCGCTGTGGGGACTTCTGACTTTTGCATGGCCCCCGATATATACATTC
TCAATAACACTGGGTCTCAGATTAACTCCGAGGTCACTCGGTATTACCTTCACTGCTCTCAAAGTCTTATTAGCCCCTTTCAACAATCTCTGACT
ACATTCCAAAGGAGTTTGACAACTATGCAGATTCAGGTGGGGGGCTTGCTTCAATTTGCTGTCCCCCTTTTTCCAACCGCAGAGAAGGACCTGT
TGGGCATTCAACTCTTGTTGAATAACTCTGAAATCTCTCTGCACCAGCTCACCGCAATGCTTGATTGCCGGGGTTTGCACAAGGATTATCTCGA
TGCACTGACTGGAATTTGTTATGATGGCATCGAGGGCTTGCTCTTCCTCGGGCTTTTCTCACTGCTCGCTGCATTGGCCTTTAGTACACTGACT
TGCGCAGGCCCTAGAGCCTGGAAATATTTCATAAATCGCGACAGGGATTATGACGATATAGATGATGATGACCCCTTTAACCCACAGGCCAGG
CGCATCGCCGCACACAATCCTACCAGAGGGCAACTTCATTCCTTTTGTTCTTACTCCTCCGGACTCGGATCACAATGCTCTTTGCAGCCTCCTT
CTCAGACCATCAGTAATGCTCCTGTCTCCGAGTATATGAATCAAGCCATCCTTTTCGGGGGAAATCCACGATACGAAAACGTACCACTTATTGG
TCGAGGATCCCCTCCCCCTACTTACAGCCCTAGCATGAGACCCACCTATATGTCTGTCGCAGACGAACATTTGCGCCACTACGAGTTCCCCTC
C
```

mTTYH3:

```
ATGGCAGGCGTAAGCTATGCTGCCCCTTGGTGGGTCTCACTTCTCCATCGCCTTCCCCACTTTGACCTGCGGTGGGAAGCCACTAGCTCACAA
TTTAGACCTGAGGACGCAGACTATCAGCAAGCACTCCTTCTTTTGGGGGCCACTGCCCTTGCTTGTCTGGCACTGGATTTGCTGTTCCTCTTGT
TCTACTCTTTCTGGCTTTGCTGCCGAAGGAGAAAAACAGACGAACACTTGGACGCCGACTGTTGTTGTACCGCCTGGTGCGTGATTATCACCAC
ATTGGTGTGTTCAGCCGGGATTGCAGTTGGTTTCTATGGGAATGGGGAAACCTCTGATGGTATCCACCGAGCTACATACAGCCTGCGGCACGC
TAATAGAACAGTAGCTGGGGTACAAGATCGAGTCTGGGATACAGCCGCTGCCCTCAATCGAACCGCCGAGCCCAACCTGCAGTCTCTTGAAAG
ACAGCTCGCCGGACGCCAGGAACCCCTGCGGGCTGTGCAACGCCTTCAAACTCTCCTTGGTACTCTTCTCGGGTATACAGCCGCTATCCCCTT
CTGGAGGAATCCAGGTGTTAGCTTGGAAGTCCTTGCTGAGCAAGTGGATTTGTATGACTGGTACAGATGGCTGGGGTACTTGGGTCTGCTTCT
CCTGGATGTGATTATTTGCCTTCTTGTGCTTGTAGGATTGATAAGGTCATCAAAAGGCATTCTTGTAGGCGTCTGTTTGTTGGGCGTACTGGCCT
TGGTTATATCATGGGGAGCCTTGGGATTGGAACTGGCTGTCAGTGTGGGCTCTAGCGATTTCTGCGTTGACCCTGATACTTTTGTGACAAAAAT
GGTGGAAGAGCATAGTGTGCTCTCTGGGGATATTTTGCAGTATTATCTGGCCTGCTCCCCCCGAGCCACTAATCCATTCCAACAGAAATTGTCT
GGTTCACATAAAGCACTGGTAGAAATGCAGGATGTGGTCGCTGAGCTGCTCAGGAATGTGCCCAGGGAGCACCCAGCTACAAAAGACCCCCT
CCTTAGAGTCCAAGAAGTCCTGAATGGTACAGAAGTCAATCTCCAGCATCTGACTGCCCTCGTAGATTGCCGATCTTTGCATCTTGACTACGTA
CAGGCTTTGACAGGCTTTTGTTACGATGGCGTTGAAGGTTTGATCTATCTTGCACTGTTCAGCTTTGTGACAGCCTTGATGTTCTCCAGTATCGT
CTGCAGTATTCCACATACTTGGCAACAAAAACGGGGCCCTGACGATGACGGAGAAGAAGAAACCGCACCAGGACCTAGGCAAGCTCACGACT
CACTTTATAGAGTCCACATGCCTTCACTGTACAGCTGTGGATCTAGCTACGGTAGCGAGGCATCCATACCTGCCGCTGCACACACAGTTAGCAA
TGCTCCCGTTACAGAGTACATGTCTCAGAACGCCAATTTTCAGAACCCTCGATGCGAGAATACACCTCTCATTGGCCGCGAATCACCTCCCCCA
TCCTATACAAGTTCCATGCGAGCAAAATACTTGGCAACTTCCCAACCCAGGCCCGATTCTAGCGGATCTGGCCAC
```
