# Supplementary material for: Structures of tweety homolog proteins TTYH2 and TTYH3 reveal a Ca2+-dependent switch from intra- to intermembrane dimerization
Source: Nat Commun. 2021 Nov 25;12:6913. doi: 10.1038/s41467-021-27283-8 (PMC8617170; doi:10.1038/s41467-021-27283-8)
Supplement: Supplementary file 3 — Reporting summary [file 41467_2021_27283_MOESM3_ESM.pdf]

nature portfolio

Corresponding author(s): Brohawn, SG

Last updated by author(s): Oct 18, 2021

Reporting Summary

Nature Portfolio wishes to improve the reproducibility of the work that we publish. This form provides structure for consistency and transparency in reporting. For further information on Nature Portfolio policies, see our Editorial Policies and the Editorial Policy Checklist.

Statistics

For all statistical analyses, confirm that the following items are present in the figure legend, table legend, main text, or Methods section.

n/a	Confirmed	
☐	☒	The exact sample size (n) for each experimental group/condition, given as a discrete number and unit of measurement
☐	☒	A statement on whether measurements were taken from distinct samples or whether the same sample was measured repeatedly
☐	☒	The statistical test(s) used AND whether they are one- or two-sided<br>*Only common tests should be described solely by name; describe more complex techniques in the Methods section.*
☒	☐	A description of all covariates tested
☐	☒	A description of any assumptions or corrections, such as tests of normality and adjustment for multiple comparisons
☐	☒	A full description of the statistical parameters including central tendency (e.g. means) or other basic estimates (e.g. regression coefficient) AND variation (e.g. standard deviation) or associated estimates of uncertainty (e.g. confidence intervals)
☐	☒	For null hypothesis testing, the test statistic (e.g. F, t, r) with confidence intervals, effect sizes, degrees of freedom and P value noted<br>*Give P values as exact values whenever suitable.*
☒	☐	For Bayesian analysis, information on the choice of priors and Markov chain Monte Carlo settings
☒	☐	For hierarchical and complex designs, identification of the appropriate level for tests and full reporting of outcomes
☒	☐	Estimates of effect sizes (e.g. Cohen's d, Pearson's r), indicating how they were calculated

Our web collection on statistics for biologists contains articles on many of the points above.

Software and code

Policy information about availability of computer code

Data collection	SerialEM 3.6, pClamp10, ChromLab 6.0
Data analysis	Relion 3.1, MotionCor2, Ctffind 4.1, Cryosparc 2, UCSF PyEM, Phenix 1.19, Coot 0.9, Molprobity 4.5, PyMOL 2.3.2, Chimera 1.12, ChimeraX 0.6, Prism 8, Topaz 0.2.5, HOLE implemented in Coot 0.9, Dali v5, JalView 2.11.1.4, Consurf 2016

For manuscripts utilizing custom algorithms or software that are central to the research but not yet described in published literature, software must be made available to editors and reviewers. We strongly encourage code deposition in a community repository (e.g. GitHub). See the Nature Portfolio guidelines for submitting code & software for further information.

Data

Policy information about availability of data

All manuscripts must include a data availability statement. This statement should provide the following information, where applicable:

- Accession codes, unique identifiers, or web links for publicly available datasets
- A description of any restrictions on data availability
- For clinical datasets or third party data, please ensure that the statement adheres to our policy

All data associated with this study will be publicly available. For the TTYH2 cis-dimer in the presence of Ca2+, the final model is in the PDB under 7RTT [http://doi.org/10.2210/pdb7RTT/pdb], the final map is in the Electron Microscopy Data Bank (EMDB) under EMD-24688 [https://www.ebi.ac.uk/emdb/EMD-24688], and the original micrograph movies and final particle stack are in the Electron Microscopy Public Image Archive (EMPIAR) database. For the TTYH2 trans-dimer in the absence of Ca2+, the final model is in the PDB under 7RTU [http://doi.org/10.2210/pdb7RTU/pdb] and the final map is in the EMDB under EMD-24689 [https://www.ebi.ac.uk/emdb/EMD-24689]. For the TTYH2 monomer in the absence of Ca2+, the final model is in the PDB under 7RTV [http://doi.org/10.2210/pdb7RTV/pdb] and the final map is in the EMDB under EMD-24690 [https://www.ebi.ac.uk/emdb/EMD-24690]. Original micrograph movies and final particle stacks for the

March 2021

TTYH2 monomer and trans-dimer are in the EMPIAR database. For the TTYH3 cis-dimer in the presence of Ca2+, the final model is in the PDB under 7RTW [http://doi.org/10.2210/pdb7RTW/pdb], the final map is in the EMDB under EMD-24691 [https://www.ebi.ac.uk/emdb/EMD-24691], and the original micrograph movies and final particle stack are in the EMPIAR database. Source data are provided with this paper.

Field-specific reporting

Please select the one below that is the best fit for your research. If you are not sure, read the appropriate sections before making your selection.

[x] Life sciences [ ] Behavioural & social sciences [ ] Ecological, evolutionary & environmental sciences

For a reference copy of the document with all sections, see nature.com/documents/nr-reporting-summary-flat.pdf

Life sciences study design

All studies must disclose on these points even when the disclosure is negative.

Sample size	No statistical tests were used to predetermine sample sizes. Sample sizes for electrophysiology were chosen based on established practice in the field and were sufficient as differences between groups were reproducible. See e.g. Li et al. Nature 2020.
Data exclusions	No data were excluded from analyses.
Replication	All attempts at replication were successful. See legends for numbers of replicates performed.
Randomization	Not applicable to our study. No predetermined control and sample groups were used.
Blinding	Not applicable to our study. No populations were preassigned to groups so blinding was not relevant.

Reporting for specific materials, systems and methods

We require information from authors about some types of materials, experimental systems and methods used in many studies. Here, indicate whether each material, system or method listed is relevant to your study. If you are not sure if a list item applies to your research, read the appropriate section before selecting a response.

Materials & experimental systems

n/a	Involved in the study	
[x]	[ ]	Antibodies
[ ]	[x]	Eukaryotic cell lines
[x]	[ ]	Palaeontology and archaeology
[x]	[ ]	Animals and other organisms
[x]	[ ]	Human research participants
[x]	[ ]	Clinical data
[x]	[ ]	Dual use research of concern

Methods

n/a	Involved in the study	
[x]	[ ]	ChIP-seq
[x]	[ ]	Flow cytometry
[x]	[ ]	MRI-based neuroimaging

Eukaryotic cell lines

Policy information about cell lines

Cell line source(s)	HEK293 GNTI- (ATCC CRL-3022), HEK293 (ATCC CRL-3216), SF9 (Expression systems 94-001F)
Authentication	We did not further authenticate the cell lines.
Mycoplasma contamination	We did not test for mycoplasma.
Commonly misidentified lines (See ICLAC register)	No commonly misidentified lines were used in our study.
